# O.4.1-4 Integrated geriatric care - a community approach to preventing falls and frailty of elderly Slovenian

**DOI:** 10.1093/eurpub/ckad133.169

**Published:** 2023-09-11

**Authors:** Tjaša Knific, Martina Horvat, Branko Gabrovec, Gregor Veninšek

**Affiliations:** National Institute Of Public Health, Slovenia; tjasa.knific@nijz.si; National Institute Of Public Health, Slovenia; tjasa.knific@nijz.si; National Institute Of Public Health, Slovenia; tjasa.knific@nijz.si; National Institute Of Public Health, Slovenia; tjasa.knific@nijz.si

## Abstract

**Purpose:**

The need for geriatric treatment is increasing with the aging of the population. Multimorbid patients with social care problems and increased need for rehabilitation and paliative care are more common. Mortality of the elderly, due to falls is 2.5 times higher in Slovenia than in the other EU countries. Until the national regulation of integrated geriatric care in Slovenian health system and in other relevant environments (community, long-term care, social care) and the establishment of appropriate, licensed postgraduate training for health professionals, it is necessary to start educating wider range of health and social care personnel, for most common geriatric syndromes such as frailty, falls and NCDs. Establishing a community approach to health is one effective way to address vulnerability of elderly population.

**Project description:**

Project Integration of geriatric care for the elderly is implementing the existing commitment from WHO Decade of healthy ageing and the Slovenian Health Care Plan until 2025. With planned activities, it will ensure equal access to quality and safe health services, integrated and comprehensive treatment and will contribute to the deinstitutionalization of the Slovenian eldest population. It will develop a unified set of tools, for the systematic identification of elderly needs, new digital tools for optimization and better communication between different levels of providers and training a wider range of new and already employed health and social care personnel, for optimal health care. Evaluation of new competency models for health care personnel, training methods and proposed clinical pathways will be carried out at the end of the project. Based on final proposals, we will establish a new approach to the system level of integrated geriatric care and bring the treatment closer to the patient's home.

**Conclusions:**

Project will also contribute to upgrading competences of physiotherapists, kinesiologists, standard health teams in Health Promotion Centers, community nurses, family medicine and internal medicine doctors and social care workers for the integrated geriatric care for the elderly. It will address especially treatment of the physical component in geriatric syndrome, such as decreased muscle strength, balance, impaired walking and frailty, at different levels of health care and communities.

